# Molecular Modeling Reveals the Novel Inhibition Mechanism and Binding Mode of Three Natural Compounds to *Staphylococcal* α-Hemolysin

**DOI:** 10.1371/journal.pone.0080197

**Published:** 2013-11-27

**Authors:** Jiazhang Qiu, Dacheng Wang, Yu Zhang, Jing Dong, Jianfeng Wang, Xiaodi Niu

**Affiliations:** 1 Department of Food Quality and Safety, Jilin University, Changchun, China; 2 Key Laboratory of Zoonosis, Ministry of Education, Institute of Zoonosis, College of Veterinary Medicine, Jilin University, Changchun, China; University of Akron, United States of America

## Abstract

α-Hemolysin (α-HL) is a self-assembling, channel-forming toxin that is produced as a soluble monomer by *Staphylococcus aureus* strains. Until now, α-HL has been a significant virulence target for the treatment of *S. aureus* infection. In our previous report, we demonstrated that some natural compounds could bind to α-HL. Due to the binding of those compounds, the conformational transition of α-HL from the monomer to the oligomer was blocked, which resulted in inhibition of the hemolytic activity of α-HL. However, these results have not indicated how the binding of the α-HL inhibitors influence the conformational transition of the whole protein during the oligomerization process. In this study, we found that three natural compounds, Oroxylin A 7-O-glucuronide (OLG), Oroxin A (ORA), and Oroxin B (ORB), when inhibiting the hemolytic activity of α-HL, could bind to the “stem” region of α-HL. This was completed using conventional Molecular Dynamics (MD) simulations. By interacting with the novel binding sites of α-HL, the ligands could form strong interactions with both sides of the binding cavity. The results of the principal component analysis (PCA) indicated that because of the inhibitors that bind to the “stem” region of α-HL, the conformational transition of α-HL from the monomer to the oligomer was restricted. This caused the inhibition of the hemolytic activity of α-HL. This novel inhibition mechanism has been confirmed by both the steered MD simulations and the experimental data obtained from a deoxycholate-induced oligomerization assay. This study can facilitate the design of new antibacterial drugs against *S. aureus*.

## Introduction

*Staphylococcus aureus* is a significant human pathogen that is capable of causing a multitude of infections, many of which are life-threatening, such as toxic shock syndrome, bacteremia, endocarditis, sepsis, and pneumonia [1]. Since 1960, methicillin-resistant *S. aureus* (MRSA) has been a worldwide challenge with limited therapeutic options for treatment [2]. For example, a 2005 survey indicated that over 18,000 deaths could be attributed to invasive MRSA infection in the United States alone [3]. Alpha-hemolysin is one of the major toxins endowed with hemolytic, cytotoxic, dermonecrotic, and lethal properties [4]. Upon binding to susceptible cell membranes, α-hemolysin monomers penetrate the plasma membrane to form cylindrical heptameric pores with a diameter of approximately 2 nm [5]. These pores result in cytoplasmic leaking and osmotic swelling, which ultimately leads to cell damage and death.

Several lines of evidence validate α-hemolysin as a significant virulence target for the treatment of *S. aureus* infection: i) most *S. aureus* strains encode *hla* (the gene encoding alpha-hemolysin) [4]; ii) it is not essential for the survival of *S. aureus*; iii) α-hemolysin is a critical virulence factor that determines the severity of *S. aureus* infections when measured in mouse models [6]–[9]; and iiii) active or passive immunization with α-hemolysin mutant protein (H35L), anti-α-hemolysin antibody, and chemicals (β-cyclodextrin derivative) that block the heptameric pore, genetically disrupt disintegrin and metalloprotease 10 (the cellular receptor of α-hemolysin), and have shown significant protection against *S. aureus* infections [10]–[13]. Furthermore, our previous study demonstrated that some compounds could significantly reduce the mortality and tissue damage of *S. aureus* pneumonia in a mouse model by preventing the self-assembly of the α-hemolysin heptamer [14]–[16].

Molecular dynamics (MD) [17]–[19] is a useful computational tool that can offer insight into specific molecular interactions between proteins and inhibitors at the atomic level. For example, in our previous reports, we demonstrated that baicalin, a natural compound, could bind to the binding sites of Y148, P151 and F153 in α-hemolysin (α-HL) using Molecular Dynamics (MD) simulations and mutagenesis assays [14]. This binding interaction inhibits heptamer formation. In addition, through Molecular Dynamics (MD) simulations and free energy calculations, we confirmed that oroxylin A (ORO) and cyrtominetin (CTM) could inhibit the hemolytic activity of α-hemolysin (α-HL) by binding with the “Loop” region of α-hemolysin (α-HL), which is different from baicalin [15], [16]. Because of the binding of ORO and CTM, the conformational transition of the critical “Loop” region from the monomeric α-HL to the oligomer was blocked. This resulted in inhibition of the hemolytic activity of the protein.

In our study, we found that three natural compounds, Oroxylin A 7-O-glucuronide (OLG), Oroxin A (ORA) and Oroxin B (ORB), which have similar structures, can suppress the hemolytic activity of α-HL at very low concentrations. The structures are different from our previously identified compounds (e.g. Baicalin and cyrtominetin) that can block the self-assembly of α-HL heptamer [14], [16]. Thus, it is reasonable to speculate that the binding sites and binding modes of Oroxylin A 7-O-glucuronide (OLG), oroxin A (ORA) and oroxin B (ORB) would be different from baicalin or cyrtominetin. In this paper, the mechanisms of these compounds on inhibiting the hemolytic activity of α-HL were investigated, this would benefit for our understanding on drug discovery that targets staphylococcal α-HL. To explore the inhibition mechanism at the new binding sites of α-HL, we have performed Ligand-residue interaction decomposition and mutagenesis assays of three of the α-HL-inhibitor complexes in an attempt to identify specific residues that are important to the binding of α-HL inhibitors. A principle component analysis (PCA) was performed to address the collective motions of free protein and complexes. Based on the principle component analysis (PCA) simulations, the motion modes of the free protein were compared with those of the complexes, which led to the conclusion that the binding of the inhibitors hides the motion of the α-HL from the monomer to the oligomer. This inhibition activity mechanism is confirmed by the relative binding free energies calculated for the complexes based on performed potential of mean force (PMF) and the available experimental data obtained from a deoxycholate-induced oligomeriazation assay. The resulting dynamic models of the inhibitors' binding and interaction within the active site are important in the design of novel anti-virulence therapeutics.

## Results

### Oroxylin A 7-O-glucuronide (OLG), oroxin A (ORA) and oroxin B (ORB) markedly inhibit the hemolytic activity of α-HL

**Figure 1:**
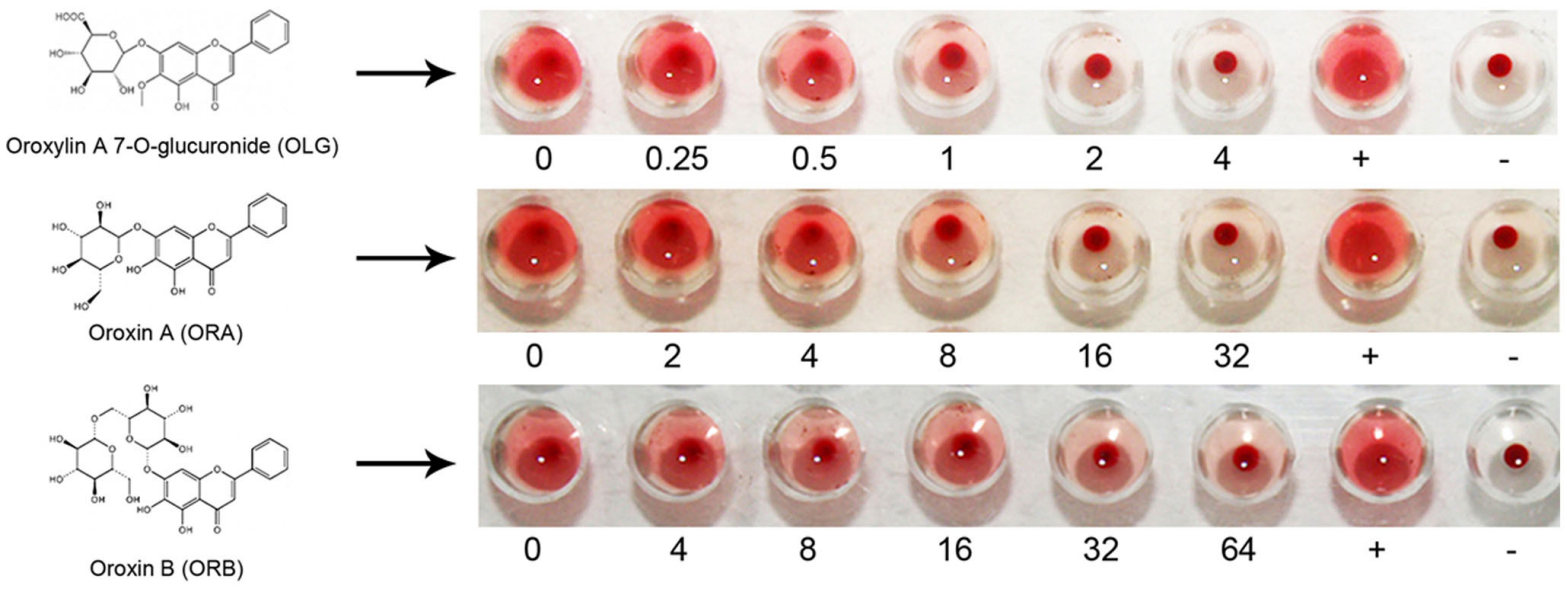
Hemolytic activity of α-HL after treating with increasing concentrations (µg/ml) of OLG, ORA and ORB. “+” indicates the positive control while “-” indicates the negative control.

Thus, α-HL has been shown to be a promising target for the development of anti-*S. aureus* agents. Screening for compounds that potentially inhibit α-HL function would facilitate the process of developing anti-α-HL drugs. In this study, we identify three compounds (OLG, ORA and ORB) that share structural similarity and could significantly suppress the hemolytic function of α-HL by screening with natural compounds (**Fig. 1** **and** **Table 1**). The 50% inhibitory concentrations were 0.73, 6.69 and 13.15 µg/ml for OLG, ORA and ORB, respectively. OLG displayed the strongest inhibitory action among these three compounds.

**Table 1:** The influence of OLG, ORA, and ORB on the hemolysis of α-HL.

Compounds	Percent hemolytic activity (%) of α-HL following treatment with increasing concentrations					
	(µg/ml) of natural compounds^a^					
OLG	0	0.25	0.5	1	2	4
	88.9±3.2	78.2±5.1	57.9±3.4*	25.4±3.7**	10.8±2.3**	5.11±2.1**
ORA	0	2	4	8	16	32
	91.4±4.6	85.8±5.7	60.5±2.7 *	40.6±1.05**	25.9±4.1**	8.3±4.4**
ORB	0	4	8	16	32	64
	87.7±4.8	67.0±5.3	47.2±4.6**	37.4±5.9**	24.4±3.6**	15.1±1.2**

a percent hemolysis, calculated by comparing with Triton X-100 treated samples.

* indicates *P*<0.05, while ** indicates *P*<0.01.

### 3D Structures of α-HL-inhibitor binding

**Figure 2:**
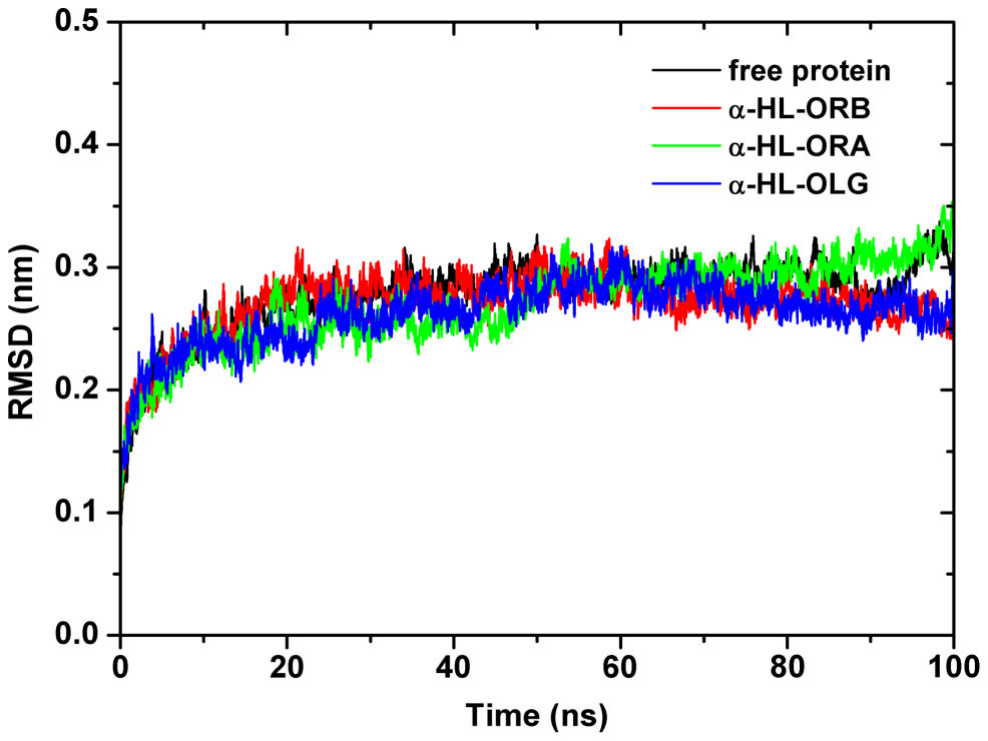
RMSD of the protein backbone, residues 1-293. The RMSD value is averaged over the free α-HL monomer and three α-HL-inhibitor systems in each replicate MD simulation.

As suggested in our previous studies [14]-[16], the experimental 3D structure of monomeric α-HL is not available; therefore, we constructed a 3D model of α-HL by homology modeling based on the NMR structure of LukF (PDB code: 1LKF), LukF-PV (PDB code: 1PVL), Gamma-hemolysin component A (PDB code: 2QK7), and LukS-PV (PDB code: 1T5R). We studied the binding of oroxin B (ORB), oroxin A (ORA), and oroxylin A 7-O-glucuronide (OLG) to the active sites of α-HL via molecular docking and molecular dynamics simulations using the AutoDock 4.0 and Gromacs 4.5.1 software packages, respectively. We simulated each α-HL-inhibitor system under the production Molecular Dynamics (MD) conditions for 100 ns to obtain sufficient number of samples. The root-mean-square deviation (RMSD) of a protein as a function of time is a good indicator of convergence in protein structure changes over the course of a simulation. The backbone root-mean-square deviation (RMSD) of the unliganded protein equilibrated to between 0.28 and 0.32 nm after ∼20 ns of simulation, as shown in **Figure 2**. Similarly, the backbone root-mean-square deviation (RMSD) of each α-HL-inhibitor system equilibrated just slightly lower, between 0.26 and 0.30 nm, over the same time frame. The average RMSD over the final 80 ns of each set of three complexes was 0.28±0.02 nm and 0.30±0.02 nm for the unliganded protein. The relative stability of each structure after the initial 20 ns provides assurance that the final 80 ns of the simulation are suitable for analysis.

**Figure 3:**
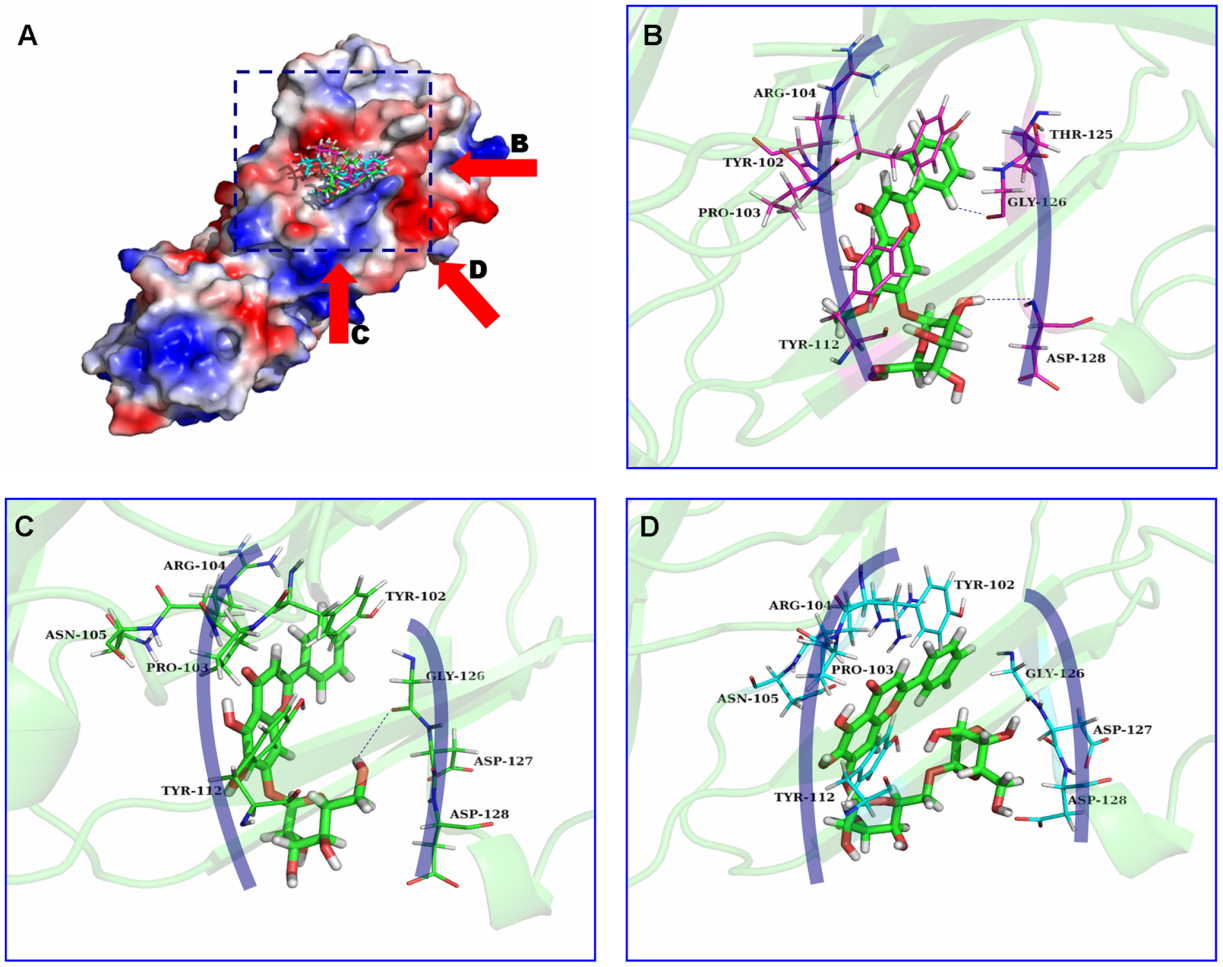
(A) Overview of the α-HL: compound complexes based on MD simulation. The arrows indicate the predicted binding mode of oroxylin A 7-O-glucuronide (OLG) with α-HL (B), oroxin A (ORA) with α-HL (C), and oroxin B (ORB) with α-HL (D). The shadow region represents the left region (L) and right region (R) of the binding cavity.

The complex structure based on the docking results was used as the initial structure of the 100-ns molecular dynamics simulations, and the preferential binding modes of the inhibitors to the α-HL were determined. The simulations show that OLG, ORA and ORB are ligands that can bind to α-HL via hydrogen bonding and the electrostatic interactions. The predicted binding modes of the OLG, ORA and ORB with α-HL are illustrated in **Figure 3A**. In detail, the binding cavities of the OLG, ORA and ORB can be divided into two categories: the right region (R) and the left region (L).

The binding model of OLG to the binding sites of α-HL (**Figure 3B**) revealed that the 4H-chromen-4-one moiety of OLG could form strong van der Waals interactions with the side chains of the Tyr102, Pro103, and Arg104 amino acids in the left region. In addition, the plane of the 4H-chromen-4-one moiety of OLG is parallel to the plane of the benzene ring of the residue Tyr112 (left region), which indicates that a strong interaction between this residue and OLG most likely exists. At the same time, the hydroxyl of the glycoside group and the hydrogen atom of the benzene ring of OLG can form two hydrogen bonds with the side chain of the Gly126 and Asp128 in the right region of α-HL. This leads to electrostatic interactions between OLG and the right region of the binding cavity. The binding mode of ORA is similar to that of OLG, as shown in **Figure 3C**. On the left region of the binding cavity, van der Waals interactions likely exist between the residues Tyr102/Pro103/Arg104/Tyr112 and ORA. On the opposing side of the binding cavity, ORA could form a hydrogen bond with the side chain of the Gly126. This leads to an electrostatic interaction between ORA and this residue. However, ORB binds with α-HL in a different manner (**Figure 3D**). In the left region of the binding cavity, ORB could also form strong van der Waals interactions with the residues Tyr102, Pro103, Arg104, and Tyr112, whereas the interaction between ORB and the residues Thr125, Gly126 and Asp128 in the right region of the binding cavity become weaker. These above-mentioned results will be confirmed by energy decomposition analysis.

**Figure 4:**
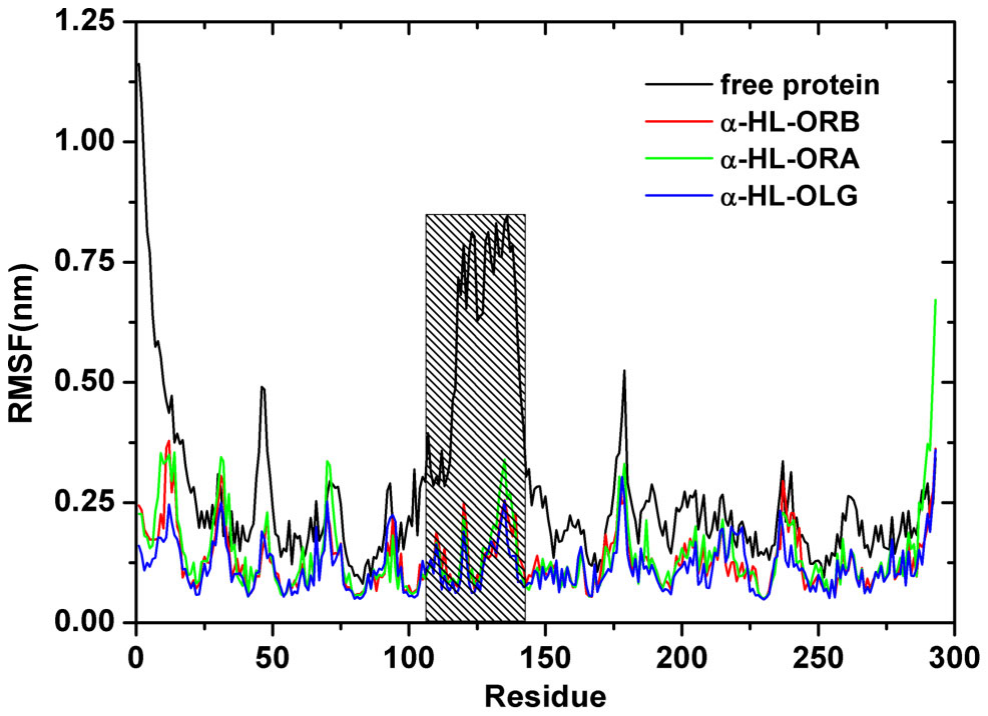
The RMS fluctuations of the whole residues in the complex and free α-HL. The region of the protein backbone exhibits different fluctuations, which are dependent on the local environment (binding with inhibitors), specifically residues 100–150. The region is highlighted with gray bars.

In contrast to RMSD, a global measurement of the protein motion, the root-mean-square fluctuation (RMSF) is a local measurement that provides a higher resolution for the details of the residue fluctuations. During the final 80 ns of the simulation, there are differences in the fluctuation patterns of the complexes and the free protein in a specific region of α-HL (**Figure 4**). This region comprised residues 100–150, which includes the majority of the binding sites (residues 101–128). All of the residues in the α-HL binding site that become bound by the inhibitors show a lower degree of flexibility (with root-mean-square fluctuation (RMSF) values less than 0.3 nm) compared to the root-mean-square fluctuation (RMSF) values calculated for the free α-HL. This indicates that these residues become more rigid after binding to the inhibitors. These results indicate that the stabilization of the α-HL binding cavity in this complex is primarily the result of the residues Tyr102/Pro103/Arg104/Tyr112 and Gly126/Asp128.

### Identification of the binding sites of the inhibitors

**Figure 5:**
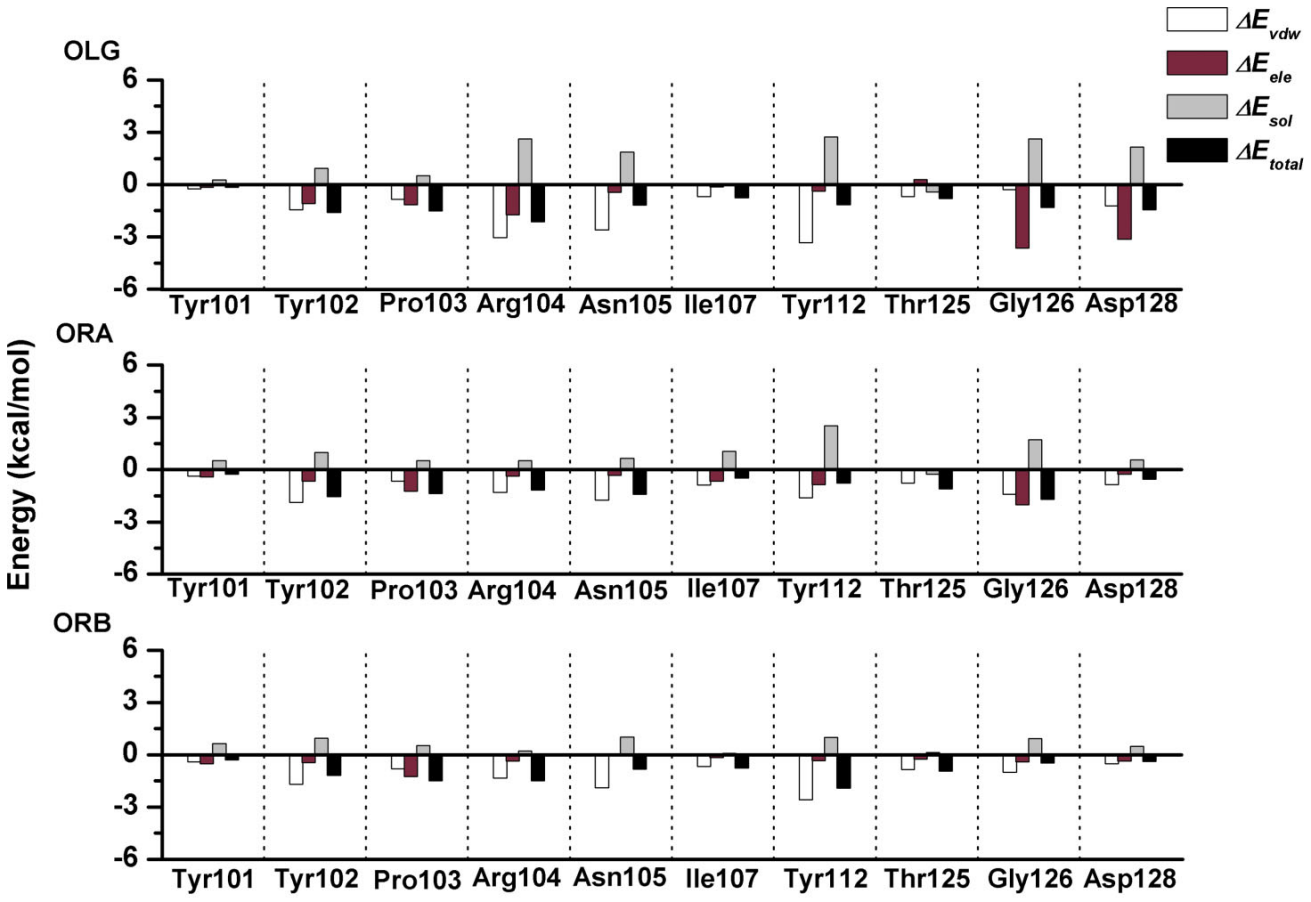
Summed energies of interaction between the inhibitors and α-HL. The histogram shows the van der Waals, electrostatic, salvation and total contributions for the complexes.

Quantification of the average energy of the interaction between each inhibitor and the specific residues in the α-HL binding site could provide an excellent indication of which residues were important to the inhibitors' binding. Therefore, ligand-residue interaction decomposition was performed for the three complexes based on the Molecular Mechanics Generalized Born Surface Area (MM-GBSA) method. The van der Waals and electrostatic contributions to the potentials of the interactions between the inhibitors and the individual residues were recorded. The inhibitors interacted with as many as 20 different α-HL residues during binding site egress; however, we plotted only the 10 strongest interactions for simplicity. The energy contributions from the selected residues are summarized in **Figure 5**.

As shown in the α-HL-OLG complex, Arg104, Asn105 and Tyr112 have the appreciable van der Waal interactions of ≤−3.0 kcal/mol with OLG because of the close proximity between these residues and the 4H-chromen-4-one moiety of OLG. In addition, Gly126 and Asp128 have the strongest electrostatic interaction, ≤−3.0 kcal/mol, with OLG, which indicates that hydrogen bonds likely exist between OLG and the protein. The binding mode of ORA is similar to that of OLG. On the left region of the binding cavity, the Tyr102, Arg104 and Asn105 have appreciable van der Waal interactions of ≤−1.60 kcal/mol with OLG. On the other side of the binding cavity, ORA had a strong interaction with the Gly126 (*ΔE_ele_* = −2.02 kcal/mol), primarily due to the electrostatic interactions. The calculations imply that ORA could also bind with both sides of the binding cavity of α-HL. However, ORB could bind with α-HL in a different manner. On the left region of the binding cavity, Tyr102, Arg104, Asn105, and Tyr112 have the appreciable *ΔE_vdw_* of −1.69, −1.35, −1.88, and −2.58 kcal/mol, respectively, which indicates that ORB can bind with the left region of the binding cavity effectively. While on the right region of the binding cavity of α-HL, the interaction between ORB and the Gly126 and the Asp128 become weaker, as shown in **Figure 5**. These results show good agreement with the data shown in **Figures 3** **and** **4**, which indicates that the residues of Tyr102, Arg104, Asn105, Tyr112, and Gly126 could be important for the binding of the inhibitors.

To verify the accuracy of this hypothesis, the total binding energy of three wild-type complexes and two α-HL mutants (R104A-HL and G126A-HL) with three inhibitors complexes were calculated using the Molecular Mechanics Generalized Born Surface Area (MM-GBSA) method. Consequently, the total binding energy was compared with the binding free energy of the complexes using the fluorescence spectroscopy quenching method[20], [21]. As shown in **Table 2**, the Molecular Mechanics Generalized Born Surface Area (MM-GBSA) calculation predicted that mutants wound bind more weakly to the inhibitors than to the wild type (WT)-HL systems with estimated *ΔG_bind_* values of −8.4, −4.2, −1.4, −8.9, −4.0, −2.3 kcal/mol for R104A-OLG, R104A-ORA, R104A-ORB, G126A-OLG, G126A-ORA, and G126A-ORB, respectively. The experimental measurement of the binding free energy, *ΔG_bind_*, also shows that the interaction between the inhibitors and the wild type (WT)-HL is high, which means that wild type (WT)-HL has a stronger ability to bind to the inhibitors, as shown in **Table 3**. Because the calculated binding free energies are in good agreement with the experimental data, we believe that the MD simulations generated reliable models for the α-HL-OLG, α-HL-ORA and α-HL-ORB complexes.

**Table 2:** The calculated energy components, the binding free energy (kcal/mol) of OLG, ORA, and ORB binding to the active site of α-HL.

Protein-inhibitors	*ΔG_bind_* (kcal/mol)	n
**WT-OLG**	9.3	1.0115
**WT-ORA**	7.4	0.9945
**WT-ORB**	3.7	1.0501
**R104A -OLG**	7.2	1.1481
**R104A -ORA**	5.1	0.9987
**R104A -ORB**	2.8	0.9963
**G126A -OLG**	7.5	1.0214
**G126A -ORA**	3.2	1.0021
**G126A -ORB**	1.9	0.9991

**Table 3:** The values of the binding free energy (*ΔGbind*) and the number of binding sites (n) of the α-HL-inhibitor systems based on the results from the fluorescence-quenching method.

Protein-inhibitors	*ΔG_total_*	*-TΔS*	*ΔG_bind_*
**WT-OLG**	−18.3±3.4	6.4±1.3	−11.9±2.2
**WT-ORA**	−15.2±2.6	5.7±1.2	−9.5±2.0
**WT-ORB**	−9.8±1.9	5.0±0.9	−4.8±1.1
**R104A -OLG**	−14.3±2.0	5.9±1.1	−8.4±1.5
**R104A -ORA**	−9.8±1.8	5.6±0.9	−4.2±0.7
**R104A -ORB**	−6.5±1.2	5.1±1.0	−1.4±0.2
**G126A -OLG**	−15.1±2.1	6.2±1.1	−8.9±1.7
**G126A -ORA**	−9.9±1.7	5.9±1.8	−4.0±1.1
**G126A -ORB**	−7.5±2.6	5.2±1.1	−2.3±0.7

### Influence on the motion of α-HL from the binding of inhibitors

**Figure 6:**
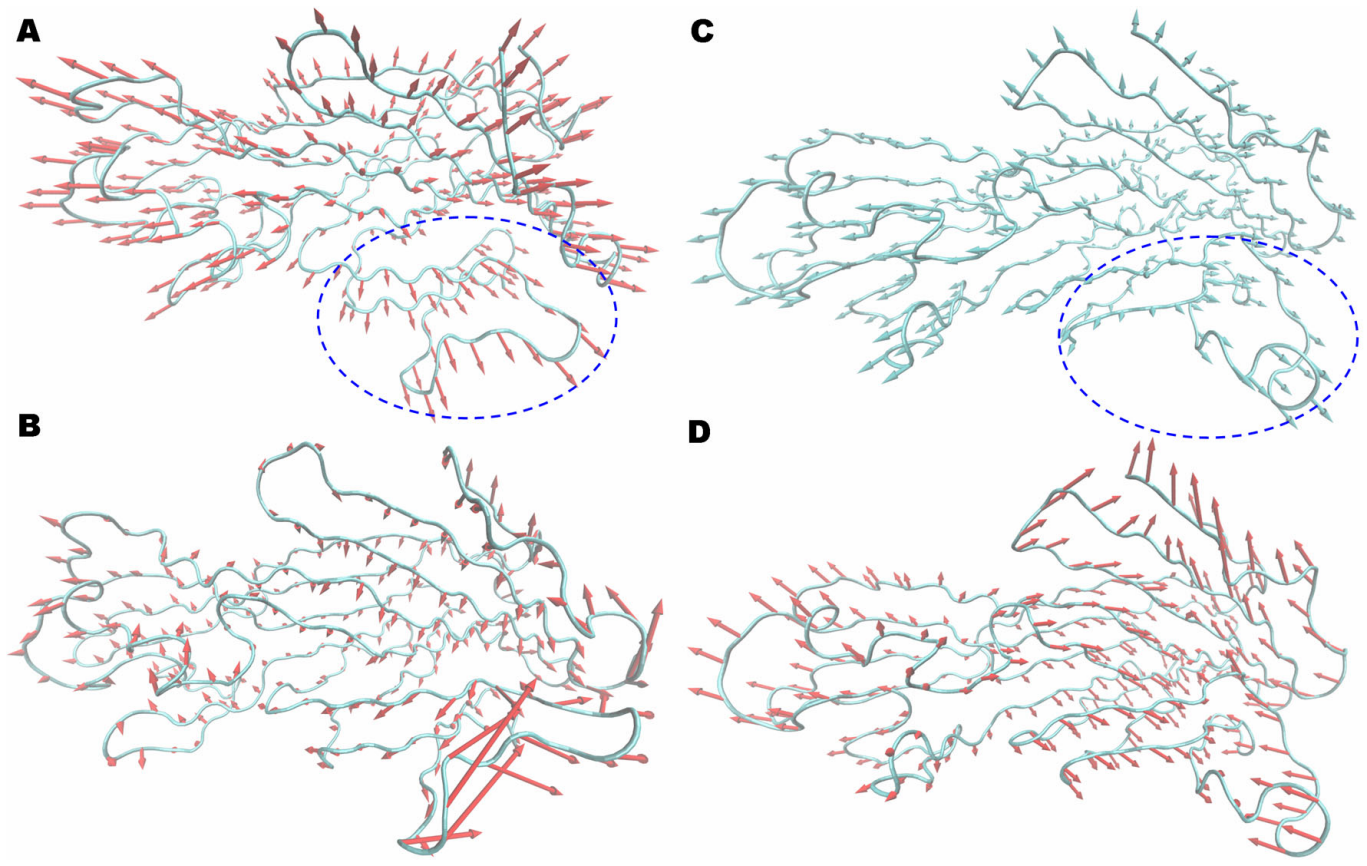
Collective motions obtained by principal component analysis on the simulation trajectory. (A) and (B) Motions corresponding to PC1 and PC2 of the unliganded α-HL, which account for 68.7 and 19.8% of the total movements, respectively. (C) and (D) Motions corresponding to PC1 and PC2 of the α-HL-OLG complex, which account for 65.4% and 18.7% of the total movements, respectively.

A principal component analysis (PCA) was performed on the MD trajectory of the free α-HL and α-HL-inhibitor complexes to identify the most significant motions of the protein in a complex or an unliganded state. The first two principal components account for 68.7% and 19.8% of the overall motion of the free protein. The first component (PC1) is composed of several motions (**Figure 6A**): as a rigid body, there is an extended motion to the entire conformation of the protein, particularly to the “stem” domain^22^ in the α-HL (represented by the dotted line in **Figure 6A**). This motion is sufficiently large to meet (but not exceed) the requirements for the conformational transition of α-HL to transition from monomeric α-HL to the oligomer. The second principal component (PC2) primarily corresponds to the slight vibration of the backbone of the protein, as shown in **Figure 6B**. We plotted only α-HL-OLG for the principle component analysis (PCA) results. The first two principal components account for 65.4% and 18.7% of the overall motion in the α-HL-OLG complex. The PC1 of the α-HL-OLG complex is composed of several motions, similar to that of th eunliganded α-HL, with the exception of the motion in the “stem” domain (represented by the dotted line in **Figure 6C**). As shown in **Figure 6C**, the motion of the “stem” domain is obviously weaker than that of the unliganded α-HL. It should be noted that the “stem” domain is also the binding site of OLG, ORA and ORB in the complexes based on previous data. Therefore, it is confirmed that the motion of the “stem” domain is restricted by the binding of the inhibitors.

**Figure 7:**
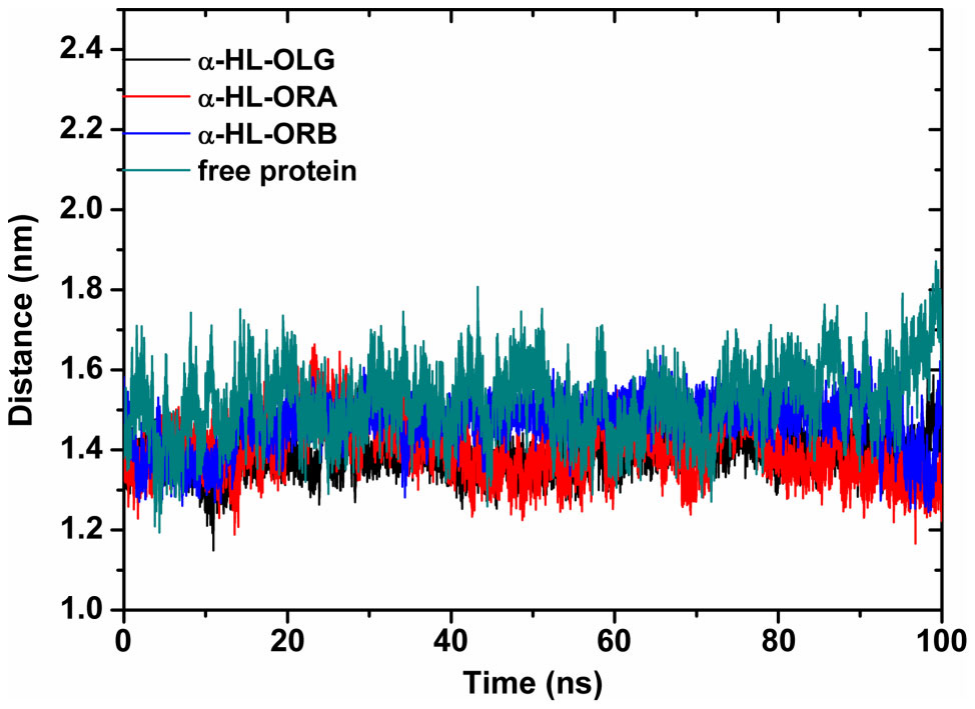
Plots of distances between the C_α_ atoms of Arg104 and Asp128 tracked through MD simulation. The average distance in OLG, ORA, ORB complexes and free protein is 1.35, 1.41, 1.45 and 1.55

In the following, the distances between the C_α_ of Arg104 and the C_α_ of Asp128 in the complexes and in the free protein were calculated during the MD simulated trajectory, as shown in **Figure 7**. The average distances for α-HL-OLG, α-HL-ORA and α-HL-ORB complexes were 1.35, 1.41, and 1.45 nm, respectively. However, the average distance for unliganded α-HL was 1.55 nm. Dynamic fluctuations in the distance between the C_α_ of Arg104 and the C_α_ of Asp128 likely indicate that the conformation of the “stem” domain is restrained when the inhibitors bind to these two residues. This is consistent with the previous results. Furthermore, we have developed a hypothesis that the extent to which the conformation of the “stem” domain is blocked is proportional to the inhibition activity of OLG, ORA, and ORB.

### The mechanism for the inhibition activity is confirmed

**Figure 8:**
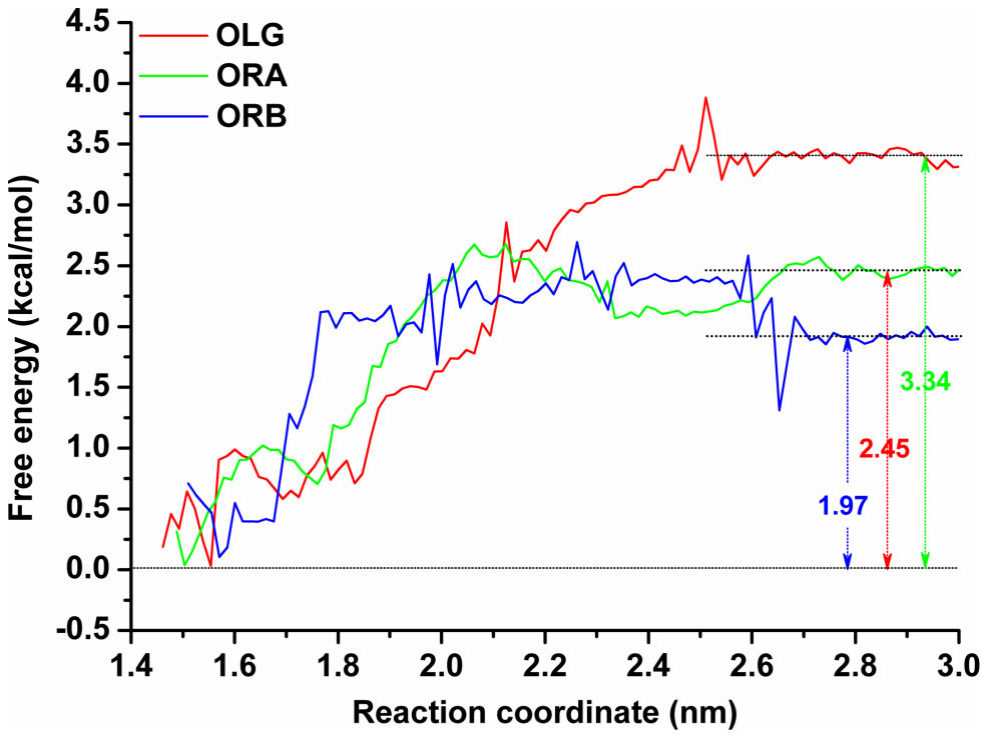
Free energy profiles determined for OLG, ORA and ORB binding with α-HL. The reaction coordinate was defined as the distance between the center of mass of all of the atoms of OLG, ORA or ORB and the center of mass of all of the atoms that belong to α-HL.

To address the above-mentioned hypothesis, we determined the performed potential of mean force (PMF) for each of the three binding complexes, including α-HL-OLG, α-HL-ORA, and α-HL-ORB, based on the data collected from the umbrella-sampling MD simulations. The performed potential of mean force (PMF) results depicted in **Figure 8** are based on the MD simulations for 10 ns windows.

Based on the calculated performed potential of mean force (PMF) profiles (**Figure 8**) we determined that the binding free energies calculated for the three complexes differ from each other. The binding free energy (*ΔG*_bind_) for the α-HL-OLG system has a value of −3.34 kcal/mol. The calculated binding free energy of the α-HL-ORA system is −2.45 kcal/mol, and the calculated binding free energy for the α-HL-ORB system is −1.97 kcal/mol. According to these results, the affinities of the three ligands binding with α-HL follow the trend of OLG>ORA>ORB. Then, based on the above-mentioned hypothesis, the inhibition activity of the three ligands should also follow the trend of OLG>ORA>ORB.

**Figure 9:**
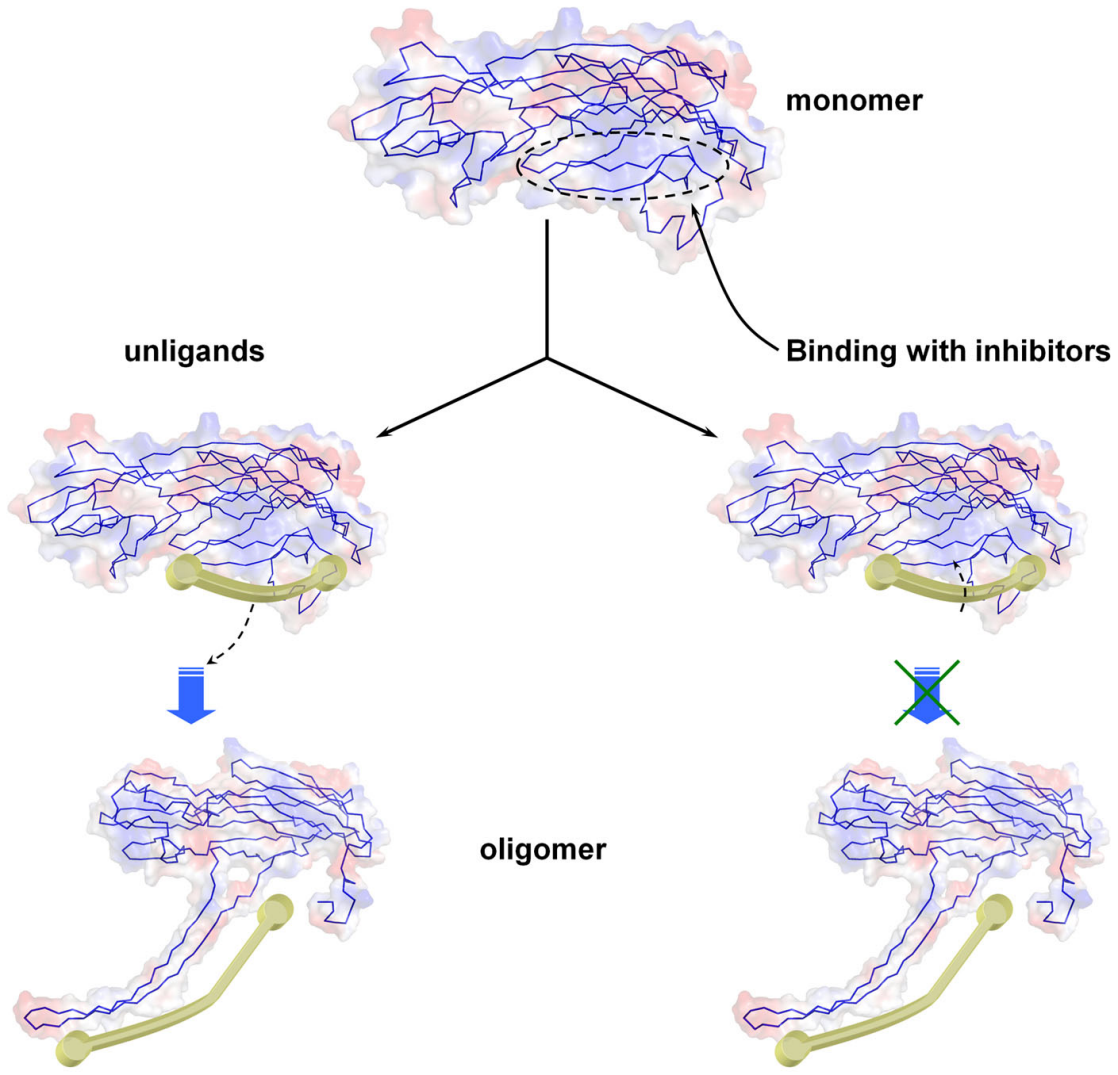
OLG (A), ORA (B) and ORB (C) prevent the deoxycholate-induced oligomerization of α-HL. α-HL was treated with 5 mM deoxycholate in the presence or absence ligands. Following sodium dodecyl sulfate (SDS)-polyacrylamide gel electrophoresis (PAGE) analysis, the proteins were detected by silver staining. (A) Lane 1, WT-HL; lane 2, R104A-HL; lane 3, G126A-HL; lane 4, WT-HL plus 2 µg/ml of OLG; lane 5, R104A-HL plus 2 µg/ml of OLG; lane 6, G126A-HL plus 2 µg/ml of OLG. (B) Lane 1, WT-HL; lane 2, R104A-HL; lane 3, G126A-HL; lane 4, WT-HL plus 16 µg/ml of ORA; lane 5, R104A-HL plus 16 µg/ml of ORA; lane 6, G126A-HL plus 16 µg/ml of ORA. (C) Lane 1, WT-HL; lane 2, R104A-HL; lane 3, G126A-HL; lane 4, WT-HL plus 32 µg/ml of ORB; lane 5, R104A-HL plus 32 µg/ml of ORB; lane 6, G126A-HL plus 32 µg/ml of ORB.

This hypothesis is confirmed by the results of the experiment, as shown in **Figure 9**. Data from a deoxycholate-induced oligomeriazation assay [22] show that the site-directed mutagenesis of R104A and G126A has no influence on the assembly of the SDS-stable oligomer, α-HL_7_. However, the formation of α-HL_7_ was inhibited when treated with OLG (2 µg/ml), ORA (16 µg/ml) or ORB (32 µg/ml), which is in good agreement with the calculated results. Furthermore, this inhibitory effect was decreased with either of the two mutants due to the weaker affinities of the three ligands binding with α-HL in the protein-ligand complexes, as shown in **Figure 9**. These findings support the following inhibition mechanism: the binding of inhibitors to the “stem” domain blocks the conformational change from monomer to heptamer, thereby decreasing the lytic activity of α-HL.

## Discussion

In this study, we performed classical MD simulations of the WT-HL and mutant binding with the OLG, ORA and ORB systems, followed by steered MD simulations of the WT-HL and the inhibitors in a solvent. Taken together, these data effectively describe the mechanism of the inhibition activity from the inhibitors and the role of the key residues around the binding site of the inhibitors.

As we all know, α-hemolysin from *S. aureus* is an important drug target for the treatment of *S. aureus* infection., It is necessary to determine what factors govern the inhibitor binding process and the inhibition specificity, including the roles of key residues around the binding site of the inhibitors, to design more specific, and thereby more effective, inhibitors for this virulence factor. Previous studies have indicated that α-HL forms transmembrane heptameric channels that cause cell damage or death [4], [22]. The structure of the heptamer has been determined using X-ray crystallography, and this structure increases our understanding of the function of α-HL. However, to date, the monomeric crystal structure of α-HL remains unknown. Therefore, to explore the process of the inhibitors binding with α-HL, the 3D structure of the monomeric α-HL in a solution was constructed using homology modeling based on the NMR coordinates of LukF, LukF-PV, Gamma-hemolysin component A, and LukS-PV, as reported in our previous studies [14]–[16]. With the 3D structure of the monomeric α-HL, the docking process and the MD simulation were completed.

One of the major findings of this study is that the simulation and the calculated binding free energy provide direct evidence for the binding sites of the complexes. In the three α-HL-inhibitor systems, the stabilization of the α-HL binding cavity is primarily the result of the residues Tyr102/Pro103/Arg104/Tyr112 and Gly126. As shown in **Figure 3**, the benzene ring plane of Tyr102 is parallel to the benzene ring planes of OLG, ORA and ORB, and Tyr102 is close to the inhibitor (the average distance between the two aromatic rings is 0.15 nm, 0.18 nm, and 0.21 nm for OLG, ORA, and ORB, respectively), which indicates a strong interaction between the residue and the inhibitors (the *ΔE_vdw_* of OLG, ORA, and ORB with Tyr102 is −1.70, −1.55, and −1.69 kcal/mol, respectively). In addition, the benzene ring plane of the Tyr112 is parallel to the 4H-chromen-4-one moiety of the inhibitors, and the average distance between the two aromatic rings is 0.19 nm, 0.21 nm, and 0.22 nm for OLG, ORA, and ORB, respectively. In this case, a strong interaction between the residue and the inhibitors is also likely to exist (the *ΔE_vdw_* of OLG, ORA, and ORB with Tyr112 is −3.0, −3.04, and −2.58 kcal/mol, respectively). However, the interactions of Gly126 and OLG, ORA, and ORB are not identical. A strong electrostatic interaction exists between Gly126 and OLG (or ORA) (the *ΔE_ele_* of OLG and ORA with Gly126 are −2.02, −3.04 kcal/mol, respectively), which likely leads to a hydrogen bond between Gly126 and OLG (or ORA), as shown in **Figure 3B**. It is noted that OLG and ORA could form a strong interaction with both sides of the binding cavity. In contrast, the electrostatic interaction between Gly126 and ORB is weaker (the *ΔE_ele_* of ORB with Gly126 is −0.40 kcal/mol), which indicates that ORB could only form a strong interaction with one side of the binding cavity.

Another important implication of this work is the mechanism of the inhibition activity. Through the analysis of the dynamic character of the α-HL binding cavity, our MD simulations demonstrate that the conformation of the binding cavity in the complexes is relatively stable when the inhibitors bind during the simulation. In contrast, the conformation in the unliganded protein is more flexible, which suggests that the ligand binding is the active inhibition force for the conformational transition for α-HL to change from the monomer to the oligomer. As shown in **Figure 7**, the distance between the C_α_ of Arg104 and the C_α_ of Asp128 in the complexes is obviously shorter than the distance in the free protein. The distances were in the following order: free protein >α-HL-ORB>α-HL-ORA>α-HL-OLG. This result implied that the flexibilities of the binding cavity in the complexes were weaker due to the binding of inhibitors.

**Figure 10:**
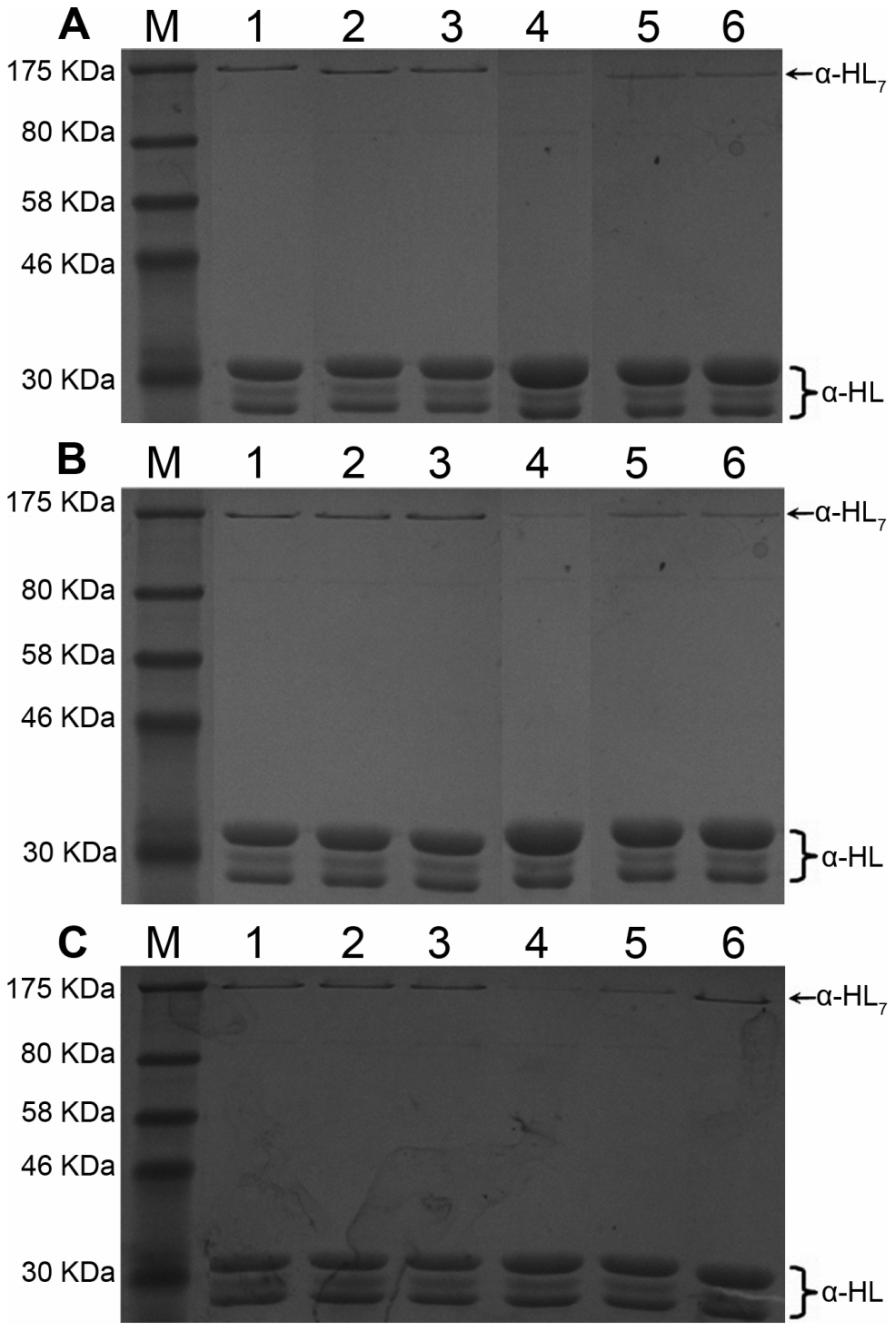
Detailed inhibitor-induced inhibition activation mechanism of α-HL. The inhibitors OLG, ORA and ORB can bind to the “stem” domain of α-HL by interactions with Tyr102/Pro103/Arg104/Tyr112 and Gly126. The ligands can form strong interactions with both sides of the binding cavity. Because of the ligands binding with the “stem” domain of α-HL, the conformational transition of α-HL from the monomeric α-HL to the oligomer was restricted, which leads to the inhibition of the hemolytic activity of α-HL.

The abundant conformations obtained in the MD simulations provide an opportunity to explore the motion properties of α-HL. Using PCA, we found a strong extended motion in the “stem” domain of the unliganded α-HL. Interestingly, compared with the structure of α-HL in the heptamer determined using X-ray crystallography [22], the “stem” domain must only complete the transition from curl to extend when α-HL changes from a monomer to a heptamer, as shown in **Figure 10**. Therefore, this conformational motion more meets the need of the conformational transition of α-HL from the monomeric α-HL to the oligomer. However, in the PCA analysis of α-HL-OLG, the motion of the “stem” domain is obviously weaker, which arises from the binding of the ligands, as shown in **Figure 8**. Then, the “stem” domain of α-HL-OLG cannot complete the transition from curl to extend, which causes the conformational transition of α-HL to be restrained, as shown in **Figure 10**. Because of the mentioned result, inhibitors can form strong interactions with both sides of the binding cavity of α-HL, which leads to the constraint of the “stem” domain.

On the basis of our MD simulation results and analyses, we propose an atomic mechanism for the ligand-induced inhibition activation of α-HL, as indicated in **Figure 10**. Initially, ligands, such as OLG, ORA, and ORB, bind to the “stem” domain of α-HL. These ligands could form strong interactions with both sides of the binding cavity by van der Waals and electrostatic interactions between the ligand and α-HL. These interactions restrict the extended motion of the “stem” domain. This “lashing force” blocks the conformational transition of α-HL from the monomeric α-HL to the oligomer, which leads to the inhibition of the hemolytic activity of α-HL. According to this mechanism, the inhibition activation of the ligands is proportional to the binding free energy in the complexes. Using steered MD simulations, the binding free energies of OLG, ORA, and ORB decrease as follows: OLG>ORA>ORB. Based on the mechanism of hemolytic inhibition, the inhibitory activity of ligands should decrease in the same order. This conclusion, which is supported by the mechanism of inhibition, is in good agreement with the experimental results. As is shown in **Figure 9**, concentrations of 2 µg/ml, 16 µg/ml, and 32 µg/ml of OLG, ORA, and ORB, respectively can inhibit the formation of α-HL_7_. However, this inhibitory effect was diminished with either of the two mutants due to the weaker affinities of the three ligands binding with α-HL in the protein-ligand complexes. Based on the above results, we believe that the mechanism of inhibition activity is reliable.

## Conclusions

In summary, the present work casts two new insights into the mechanism of inhibition activity at the atomic level by using conventional MD simulations and steered MD simulations. The results of these simulations allow for the following conclusions:

1. The inhibitors OLG, ORA, and ORB can bind to the “stem” domain of α-HL through van der Waals and electrostatic interactions. Through interactions with Tyr102/Pro103/Arg104/Tyr112 and Gly126 the ligands can form strong interactions with both sides of the binding cavity.

2. Due to the binding of the ligands to the “stem” domain of α-HL, the conformational transition of α-HL from monomeric α-HL to the oligomer was restricted. This restriction leads to the inhibition of the hemolytic activity of α-HL.

The proposed mechanism for the ligand-induced inhibition activation of α-HL could provide clues for studying the mechanism for the hemolytic inhibition of other hemolysins and for designing new and more effective antibacterial agents.

## Materials and Methods

### Hemolysis assay

The hemolysis assay was performed as previously reported. Briefly, 100 µl of purified α-hemolysin with a concentration of 500 ng/ml (Sigma-Aldrich) was preincubated in 96-well plates in the presence of OLG, ORA, and ORB at 37°C for 10 min. Then 100 µl (5×10^6^ cells/ml) of defibrinated rabbit erythrocytes was added to each well. The plates were further incubated at 37°C for 20 min. Triton X-100 (1%) served as the positive control, while PBS was used as the negative control. Following centrifugation, the supernatants were removed, and the optical density was measured at 450 nm. The percent hemolysis was calculated by comparing the supernatant reading from an equivalent number of cells that had been lysed by the Triton X-100.

### Site-directed mutagenesis of α-hemolysin

The ORF of *hla* (encoding α-hemolysin) was amplified from the *S. aureus* 8325-4 genome DNA. The primers were 5′-CGCGGATCCGCAGATTCTGATATTAATATTAAAAC-3′ (forward) and 5-′CCGCTCGAGTTAATTTGTCATTTCTTCTTTTTC-3′ (reverse). The fragment was cloned into a pGEX-6p-1 vector (Merck Biosciences) using the *BamHI* and the *XhoI* restriction sites. The resulting plasmid was designated as pGEX-6P-1-*hla*.

Plasmids encoding mutant α-hemolysin were constructed using the QuikChange site-directed mutagenesis kit (Stratagene, La Jolla, CA, USA) with plasmid pGEX-6P-1-*hla* as the template. The mutagenic primers for R104A were: 5′-CTGATTACTATCCAGCGAATTCGATTGATAC-3′ (forward) and 5′-GTATCAATCGAATTCGCTGGATAGTAATCAG-3′ (reverse). The mutagenic primers for G126A were 5′-CAACGGTAATGTTACTGCGGATGATACAGGAAAAATTG-3′ (forward) and 5′-CAATTTTTCCTGTATCATCCGCAGTAACATTACCGTTG-3′ (reverse). The underlined text indicates the codons that are to be changed.

Plasmids encoding wild type α-hemolysin and mutant α-hemolysin were transformed into *E. coli* strain BL21 (DE3). For protein production, 10 ml of the overnight culture of the *E. coli* strain harboring appropriate plasmids was transferred into 1000 ml fresh LB medium (100 µg/ml of ampicillin) and was grown until the OD_600_ value reached 0.6–0.8. IPTG was added to a final concentration of 0.2 mM. The culture was further incubated in a shaker at 16°C for 12 h. Bacteria cells were then harvested by centrifugation at 5,000 rpm (10 min at 4°C), resuspended in lysis buffer (1×PBS, 1 mM DTT and 1 mM PMSF), and homogenized by sonication. The soluble fraction was collected by centrifugation at 10,000 rpm. The GST-fused proteins were purified using glutathione Sepharose 4B beads (GE Amersham) and were digested with thrombin (Sigma-Aldrich) at 4°C overnight. The eluant was desalted using a Sephadex G-25 column (GE Healthcare). The desalted sample was purified using a Resource S 1 mL column (GE Healthcare) and was further purified using a Superdex 75 16/60 column (GE Healthcare). The purified proteins were analyzed using SDS-PAGE.

### Oligomerization assay

Reaction mixtures (50 µl) contained 5mM deoxycholate, 0.5 mg/ml WT α-HL and indicated concentrations of OLG, ORA, or ORB. Reaction mixtures were incubated at 22°C for 20 min. And reaction was stoped with 5×SDS loading buffer and boiling. 25 µl of reaction mixtures were subjected to sodium dodecyl sulfate-polyacrylamide gel electrophoresis (SDS−PAGE) followed by silver staining using the silver PlusOne staining kit (GE Healthcare) according to the manual.

### Starting Structures for Simulations

The 3D structure of the monomeric α-HL in solution was constructed using homology modeling based on the NMR coordinates of LukF (PDB code: 1LKF) [23], LukF-PV (PDB code: 1PVL) [24], Gamma-hemolysin component A (PDB code: 2QK7) [25], and LukS-PV (PDB code: 1T5R) [26], as reported in our previous work [14]. The geometry of the inhibitors was optimized at the B3LYP/6-31G^*^ level using the Gaussian 03 program.

Subsequently, to obtain the starting structure of three simulation systems for molecular dynamics (MD) simulations, the standard docking procedures for a rigid protein and three flexible ligands, oroxin B (ORB), oroxin A (ORA), and oroxylin A 7-O-glucuronide (OLG), were performed with AutoDock 4.0. The detailed process of the docking was reported in our previous work [14].

### MD Simulations

All of the simulations and the analysis of the trajectories were performed with Gromacs 4.5.1 software package [27] using the Amber Parm99sb force field and Amber 10 software package with the TIP3P water model [28]. The α-HL-inhibitor systems were first energy relaxed with 2000 steps of steepest-descent energy minimization followed by another 2000 steps of conjugate-gradient energy minimization. The systems were then equilibrated by a 500 ps of MD run with position restraints on the protein and ligands to allow for relaxation of the solvent molecules. The first equilibration run was followed by a 100 ns MD run without position restraints on the solute. The first 20 ns of the trajectory were not used in the subsequent analysis to minimize convergence artifacts. The equilibration of the trajectory was checked by monitoring the equilibration of quantities, such as the root-mean-square deviation (rmsd) with respect to the initial structure, the internal protein energy, and fluctuations calculated for different time intervals. The electrostatic term was described with the particle mesh Ewald algorithm. The LINCS [29] algorithm was used to constrain all bond lengths. For the water molecules, the SETTLE algorithm [29] was used. A dielectric permittivity, ε = 1, and a time step of 2 fs were used. All atoms were given an initial velocity obtained from a Maxwellian distribution at the desired initial temperature of 300 K. The density of the system was adjusted during the first equilibration runs at NPT condition by weak coupling to a bath of constant pressure (*P_0_* = 1 bar, coupling time τ*_p_* = 0.5 ps) [30]. In all simulations, the temperature was maintained close to the intended values by weak coupling to an external temperature bath with a coupling constant of 0.1 ps. The proteins and the rest of the system were coupled separately to the temperature bath. The structural cluster analysis was carried out using the method described by Daura and co-workers with a cutoff of 0.25 nm [30]. The MD conditions for the free a-HL protein are the same as those for the a-HL-inhibitor complexes.

The inhibitors parameters were estimated with the antechamber programs [29] and AM1-BCC partial atomic charges from the Amber suite of programs [31]. Analysis of the trajectories was performed using VMD, PyMOL analysis tools and Gromacs analysis tools.

### Steered MD Simulations

To explore the free energy profiles for the processes of the inhibitors binding with α-HL, Potential of Mean Force (PMF) calculations were performed using umbrella sampling of the MD simulations [32]. In each case, the pulling simulation was performed in quintuplicate. The starting configurations and velocities for the pulling simulations were taken at 100ps intervals. For each of the three complex structures, the total number of windows was approximately 30 and relied on the starting structure of each system. The direction of the unbinding vector was dynamically defined for each individual steered simulation based on the center-of-mass of the steered group and the center-of-mass of the active site entrance opening. The magnitude of the unbinding vector is automatically normalized in Gromacs. All of the constant-velocity steered MD simulations were implemented with a spring force constant of 1000 kJ mol^−1^ nm^−2^ and a speed of 0.01 nm ps^−1^.

### Principle Component Analysis

Principle component analysis (PCA) enables isolation of the essential subspace from the local fluctuations via the calculation of a set of eigenvectors that describe correlated motions of atoms within the MD simulation [33]. PCA was performed to address the collective motions of the α-HL-inhibitor complexes using the positional covariance matrix, *C*, of the atomic coordinates and its eigenvectors. PCA was performed with the Gromacs 4.5.1 module, and the trajectories were obtained from the previous MD simulations.

### Ligand-residue interaction decomposition

The interactions between the inhibitors and each residue in the binding site of α-HL are analyzed using the Molecular Mechanics Generalized Born Surface Area (MM-GBSA) decomposition process applied in the MM-GBSA module of Amber 10. The binding interaction of each ligand-residue pair includes three terms: the Van der Waals contribution (*ΔE_vdw_*) and the electrostatic contribution (*ΔE_ele_*). We chose a total of 100 snapshots to be captured at an evenly spaced interval over the last 80 ns on the MD trajectory.

### Fluorescence-quenching assay

The fluorescence-quenching method was used to measure the binding constants (*K_A_*) of the chemical compounds to the binding site of the WT α-HL and the mutant α-HL. The binding constants were converted to the binding energy using the equation, *ΔG_bind_* = RTln*K_A_*. Fluorescence spectrofluorimetry measurements were performed via the Horiba-Jobin-Yvon Fluorolog 3-221 spectrofluorometer (Horiba Jobin-Yvon, Edison, NJ). The measurements were acquired using 280 nm excitation with a 5 nm band-pass and 345 nm emissions with a 10 nm band-pass.

### Statistical analysis

The experimental results were analyzed by SPSS 12.0. An independent Student's *t*-test was applied to determine statistical significance, a P value less than 0.05 was considered to be statistically significant.
